# Supplementary material for: Novel Chaphamaparvovirus in Insectivorous Molossus molossus Bats, from the Brazilian Amazon Region
Source: Viruses. 2023 Feb 22;15(3):606. doi: 10.3390/v15030606 (PMC10054343; doi:10.3390/v15030606)
Supplement: Supplementary file 1 [file viruses-15-00606-s001.zip › viruses-2191982-supplementary.pdf]

FIGURES

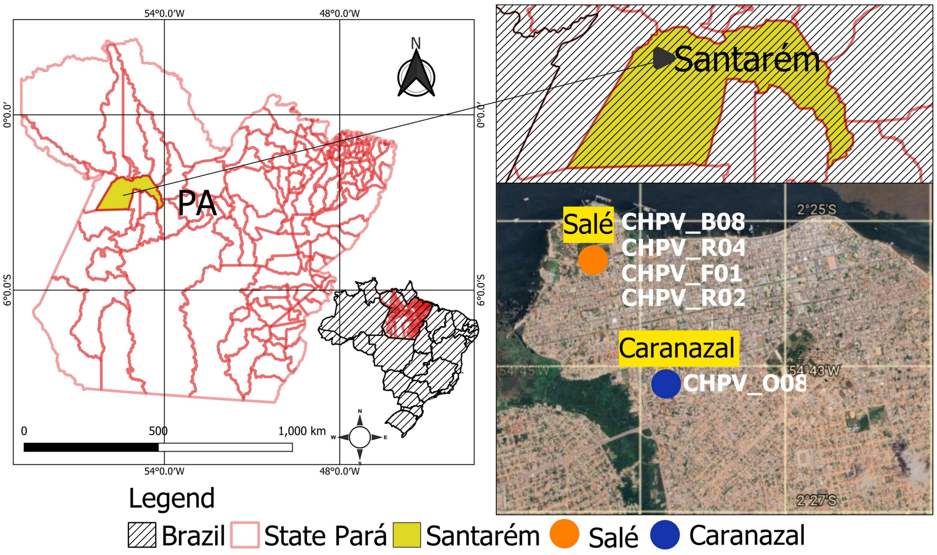

Figure S1. Map showing the locations where bats were captured. The state of Pará is indicated in red. The municipality of Santarém is indicated in yellow. Orange and blue circles indicates the locations (Salé and Caranazal) of captures in the northeastern of Santarém. THEQGIS Geographic Information System software (https://www.qgis.org accessed on 29 December 2022) and IBGE data (https://www.ibge.gov.br accessed on 29 December 2022) was used to build the map.

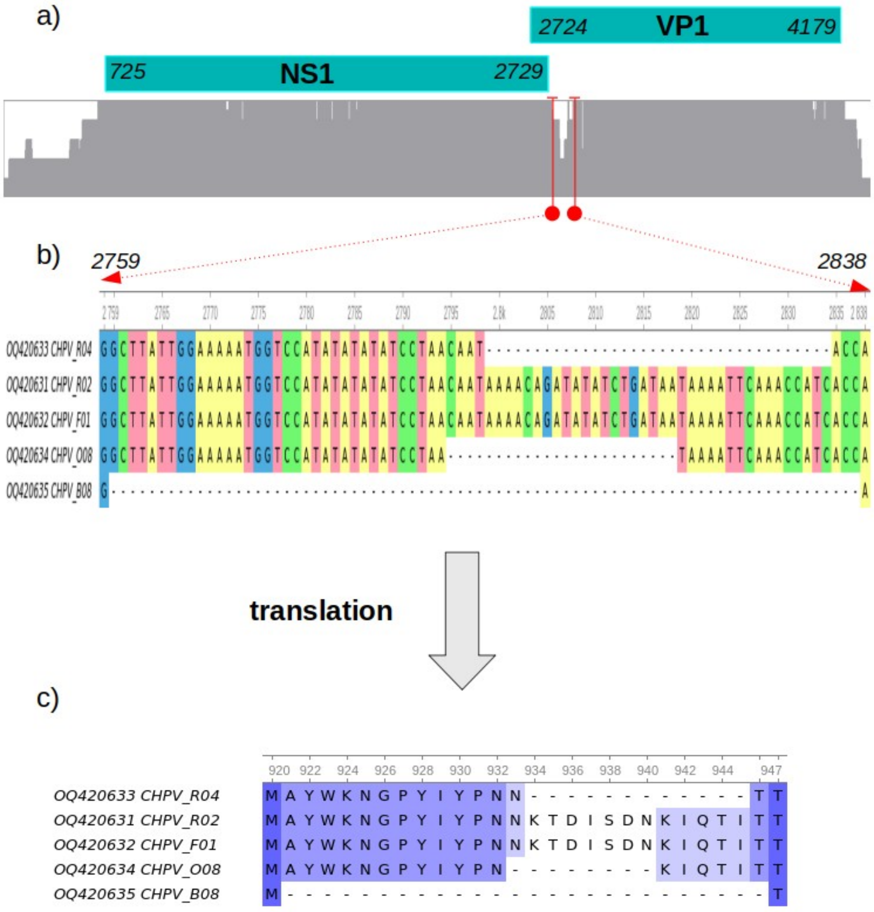

Figure S2. Alignment of Molossus CHPV. (**A**) Diagram showing the nucleotide similarity of CHPV identified in *Molossus molossus* in Brazil. Areas with high level of identity are indicated in gray while low identity is shown in white color. Above the diagram the location of NS1 and VP1 proteins are also indicated. (**B**) Region between NS1 and VP1 containing gaps in some sequences. (**C**) Translated version of the region in the alignment with gaps.

TABLE

Table S1. Virus families identified in Brazilian Bats[1].

Virus family	number	percentage
Adenoviridae	3	0,40%
Anelloviridae	18	2,38%
Astroviridae	6	0,79%
Circoviridae	29	3,84%
Coronaviridae	160	21,19%
Genomoviridae	7	0,93%
Hantaviridae	7	0,93%
Hepadnaviridae	1	0,13%
Herpesviridae	11	1,46%
Orthomyxoviridae	2	0,26%
Papillomaviridae	2	0,26%
Paramyxoviridae	39	5,17%
Parvoviridae	3	0,40%
Peribunyaviridae	1	0,13%
Polyomaviridae	2	0,26%
Reoviridae	4	0,53%
Rhabdoviridae	458	60,53%
Smacoviridae	2	0,26%

1)According to the Database of bat-associated viruses (http://www.mgc.ac.cn/cgi-bin/DbatVir/main.cgi?func=map, accessed on 9 February 2023)
